# Prevalence of Iron Deficiency Anemia among University Students in Hodeida Province, Yemen

**DOI:** 10.1155/2018/4157876

**Published:** 2018-04-23

**Authors:** Abdullah Ahmed Al-alimi, Salem Bashanfer, Mohammed Abdo Morish

**Affiliations:** Department of Medical Laboratory, Faculty of Medicine and Health Sciences, Hodeida University, Al Hudaydah, Yemen

## Abstract

**Background:**

Iron deficiency anemia (IDA) is one of the most common types of nutritional anemia in the worldwide and considered a major public health problem in developing countries especially in Yemen. Therefore, this cross-sectional study was conducted to determine the prevalence and risk factors of IDA among apparently healthy Yemeni students at Hodeida University.

**Method:**

Five hundred blood samples (326 males and 174 females) were collected randomly from medical students at Hodeida University. Participants were subjected to different tests including complete blood counts (CBC), serum ferritin (SF), serum iron (SI), and total iron binding capacity (TIBC). Moreover, a questionnaire was designed to collect demographics, food and drink habits, and socioeconomic status.

**Result:**

The overall prevalence of IDA was 30.4%  (*n* = 152), of whom 54.00% were females (*n* = 82) and 46.0% were males (*n* = 70). Students aged 20–22 years were found more anemic with prevalence 59.2% than students aged 17–19 years (25.0%) and 23–25 years (15.8%). Statistical analysis showed regularly having breakfast had significant (*p* < 0.001) role in preventing development of IDA compared with irregularly having breakfast. Infrequent consumption of vegetables/fruits; meat, fish, chicken; tea drinking; low household income; smoking and khat* (Catha edulis)* chewing showed a significant role (*p* < 0.001) in provoking of IDA, whereas consumption of coffee and cola showed insignificant influence (*p* = 0.585; *p* = 0.513) on IDA.

**Conclusion:**

This study revealed that the majority of university students, especially females, have IDA that might become worse by malnutrition, lifestyle habits, and lack of awareness. Our results suggest that IDA can be prevented by providing proper knowledge on the healthful diet, improved lifestyle, and harmful effect of IDA to the students.

## 1. Introduction

Anemia is a major public health problem in the worldwide with prevalence of 43% in developing countries and 9% in developed nations [1]. It is widespread in individuals at any stage of life, although pregnant-reproductive women and young children are most susceptible, which may increase the risk of impaired cognitive and physical development and increased mortality and morbidity rate [2].

Despite its multifactorial etiology, anemia might be nutritional (iron, folic acid, and vitamin B12), inherited (thalassemia and sickle cell), environmental pollutants (lead), infectious (malaria), socioeconomic (low maternal level of education and low household income), demographic factors (age and gender), autoimmune (hemolytic anemia), malabsorption (achlorhydria), and chronic (cancer); iron deficiency anemia IDA is the most common cause of anemia [3]. According to WHO report in 2001, around two billion individuals in the worldwide have been estimated to suffer from anemia with 50% of all anemia was documented to IDA [4].

Until today, IDA is still the most prevalent and common type of micronutrient deficiency in the developing countries [5], which results from long-term negative iron imbalance. Usually, deficiency of iron develops gradually and does not have clinically apparent symptoms until anemia becomes severe [6].

The etiology of IDA during puberty might be due to increased iron demand/loss or decreased iron intake, chronic blood loss, iron malabsorption (celiac disease), pregnancy, or parasitic infection (helminthiasis), which may lead to decreased intellectual and work performance and learning difficulties [6, 7]. Poor activity, mental, and educational performances among children that have strong relations with IDA may also continue into adulthood and cause low work efficiency which has effects on the economic productivity [3].

In the Middle East region, the reported prevalence of IDA in rural and urban people varies from 17 to 70% among preschool children; 12.6–50% among school children; 14–42% among adolescents; and 11% to more than 54% among pregnant women [3, 8, 9].

Yemen is one of the poorest countries in the Middle East. It has a very high population growth, severe urban-rural imbalances, food and water scarcity, female illiteracy, widespread poverty, and economic stagnation. According to UN agencies, about half of Yemen's population of about 26.8 million lives below the poverty line [10].

Previous studies have shown that the prevalence of IDA in Yemen varies between 73.5% and 81% among children and pregnant women, respectively [3, 11]. Most of the previous studies on anemia in Yemen were conducted on school children and pregnant women and their predictive factors. Yet there is no information about the prevalence and risks factors of IDA among Yemeni adolescents in educated communities at the university stage. Consequently, the current study aims to determine the prevalence and risk factors of IDA among apparently healthy Yemeni medical students at Hodeidah University, Yemen.

## 2. Methodology

### 2.1. Study Design

This a cross-sectional study designed to determine the prevalence among university students by analyzing blood samples to measure serum iron (SI), serum ferritin (SF), total iron binding capacity (TIBC), and complete blood pictures (CBC). Moreover, a questionnaire survey was conducted to participants to evaluate their health condition and lifestyle and social habits. The survey was conducted by Faculty of Medicine and Health Sciences, University of Hodeidah, between March and June 2017.

### 2.2. Study Subjects

The present study included 500 medical students randomly selected between the ages of 17 and 25 years and excluded female students with heavy and clotted menstrual cycle. The protocol of this study was approved by Faculty of Medicine and Health Sciences, University of Hodeida, Yemen. Questionnaires were administered randomly to each student to survey dietary drink habits, socioeconomic status, demographic information, and medical history (blood disorder). Participants were informed about the objectives of the study and experiments protocol.

### 2.3. Data and Samples Collections

The questionnaire was designed to collect demographic and socioeconomic information of the participants which includes household income (low < 50,000 YER; fair 50,000–200,000 YER; and good > 200,000 YER) and regular or irregular breakfast intake with tea or juice. The types of foods taken (fruits, vegetables, meat, fish, and chicken) were classified into the following: no, infrequently (<2 servings/week), and frequently (>2 servings/week); tea consumption: no, within or after meal, and frequently (>4 times/day); coffee and cola consumption (yes or no/week) after meal; fitness, khat (*Catha edulis*) chewing, and smoking habit defined as yes or no. Moreover, participants medical history included blood disorders or any type of anemia were classified as yes or no.

Five ml of venous blood was collected from each student and divided into two tubes and 2 ml was drawn into K3EDTA tubes to measure hematological parameters, whereas 3 ml was drawn into a plain tube with no anticoagulant to measure serum iron (SI), serum ferritin (SF), and total iron binding capacity (TIBC); biochemical tests were performed to samples with low Hb and MCV based on WHO guidelines [12] to confirm the diagnosis of IDA.

### 2.4. Hematological and Biochemical Parameters

Hemoglobin (Hb), red blood cell count (RBC), hematocrit (Hct), mean corpuscular volume (MCV), mean corpuscular hemoglobin (MCH), mean corpuscular hemoglobin concentration (MCHC), white blood cell count (WBC), red cell width distribution (RWD), and platelets were determined and measured using hematology analyzer Sysmex KX-21N.

Students with Hb levels lower than cut-off values were considered to be anemic if Hb < 13.0 g/dl, MCV < 76 fl, and RWD > 14.5% for male and Hb < 12.0 g/dl, MCV < 76 fl, and RWD > 14.5% for female.

SF (Omega Diagnostics), SI (QCA), and TIBC (QCA) were measured according to manufactured kit manual.

Students who had SF < 15 *μ*g/L, Hb < 13 g/dl, SI < 10 *μ*mol/L, and TIBC ≥ 68 *μ*mol/L were defined as IDA.

### 2.5. Statistical Analysis

Statistical analysis of the data was achieved using Statistical Package for Social Sciences (SPSS) version 18. All quantitative variables were examined for normality by Shapiro-Wilks test before analysis. Continuous variables were presented as mean and standard deviations. We used independent sample *t*-test and one-way analysis of variance (ANOVA) to compare the mean and median proportions between IDA and nonanemic students for parameters such as SI, SF, TIBC, hemoglobin, and MCV. Differences between proportions were considered statistically significant if 95% CI did not overlap. In categorical variables, percentages and frequency counts were presented using cross-tabulation test. Pearson's chi-square test was used to investigate the association between the dependent variables (IDA) and the independent variables were grouped as socioeconomic information, dietary, drinks, and chewing khat habits of students.

## 3. Results

### 3.1. Prevalence of Anemia among Students

A total number of 500 blood samples were screened for anemia. Cross-tabulation was performed to describe the association between IDA and age, gender, foods and drink habits, household income, and social habits.

Table 1 shows the overall prevalence of IDA by age and gender; 30.4% students were found anemic, of whom 47.1% were female (*n* = 82) and 21.5% were male (*n* = 70). Also the higher prevalence of IDA (59.2%) was found among students aged 20–22 years compared to students aged 17–19 (25.0%) and 23–25 (15.8%) years which revealed that the prevalence of IDA was decreasing with increase of students' age. Similarly, in the case of nonanemic students, the older age group was found to be 69.8% in 20–22 years followed by other two age groups (17–19 years: 10.4%; 23–25 years: 19.8%). Moreover, the mean of hemoglobin level for IDA male and female students was found to be 12.00 g/dl (SD ± 0.80) and 10.82 g/dL (SD ± 0.63) with low statistically significant (*p* < 0.001) compared to nonanemic male (14.41 g/dl; SD ± 0.84) and nonanemic female students (13.10 g/dl; SD ± 0.60). Similarly, the median of SF, SI, and TIBC for IDA male and female students had low significantly level compared to nonanemic male and female students groups (*p* < 0.001).

**Figure 1:**
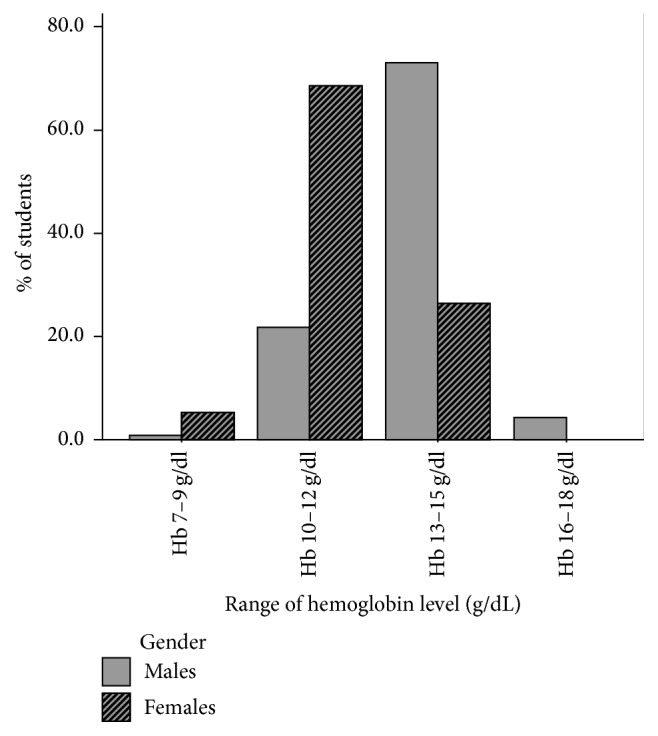
Percentage of hemoglobin concentrations for male and female students.

Figure 1 illustrates that, out of 326 male students, 73.0% had hemoglobin levels in the range of 13–15 g/dL, 21.8% in the range of 10–12 g/dL, 0.9% in the range of 7–9 g/dL, and 4.3% in the range of 16–18 g/dL. However, 68.4% of female students had hemoglobin range 10–12 g/dL, 26.4% in the range of 13–15 g/dL, and 5.2% in the range of 7–9 g/dL. Also, it was found that no female students were found with hemoglobin range 16–18 g/dL.

### 3.2. Risk Factors Enhance the Prevalence of IDA Students

Table 2 shows the association between IDA and different parameters: regular and irregular breakfast intake; dietary habits which include food (frequency intake of fruits, vegetables, meat, and fish); coffee and cocoa consumption directly after meal per week (yes or no); tea consumption (no and within and after meals, >4 caps/day); socioeconomic factors (family income, fitness, khat chewing, and smoking). Depending on the analysis in the present study, an important relationship between IDA and breakfast, drinks, weekly intake of meat, vegetables, and fruits in students at university stage was demonstrated. It was found that students who had a regular breakfast intake (76.7%) or frequently consume fruits and vegetables (46.6%) and red meat (32.5%) per week had a better iron status (nonanemic) than students who had irregular breakfast intake or insufficient portions of fruits and meat. Indeed, the prevalence of IDA was more significant among students who had irregular breakfast intake (59.2%); no (30.9%) or infrequent vegetables/fruits intake (50.7%); no (45.4%) or infrequent meat intake (36.2%) than nonanemic group who had irregular breakfast (23.3%), no (16.7%) or infrequent fruits intake (36.8%), and no (22.1%) or infrequent meat intake (45.4%). The present study further showed that students of families with low household monthly income < YER 50,000 (1 USD = 450 YER) had significant (*p* < 0.001) related factor which contributed to higher prevalence of IDA (34.2%) compared to their nonanemic counterparts (7.8%). Moreover, the prevalence of IDA was significant among students who had regular tea daily intake > 4 caps/day (37.5%) and direct intake after meal (39.5%) compared to nonanemic group, 11.5% and 29.3%, respectively. Astonishingly, there was no statistically significant difference between IDA and non-IDA students with respect to drinking coffee or cola and fitness. Additionally, there were statistically significant differences among nonanemic (47.1%) (21.8%) compared to IDA students (25.0%) (2.6%), with respect to khat chewing and smoking. Regarding awareness and unawareness of anemia among the university students, among IDA students, it was found that high significantly proportion [74.3% (113/152); *p* = 0.003] of students were unaware and 25.7% (39/152) were aware about anemia whereas 60.3% (210/326) and 39.7% (138/326) of nonanemic students were unaware and aware about anemia, respectively.

## 4. Discussion

Iron deficiency anemia is the most nutritional anemia in developed countries and become a significant health burden in the world. Many studies have demonstrated the association of IDA with impaired cognitive performance and impaired work productivity in adults [13, 14]. Previous studies have been shown the prevalence of iron deficiency anemia (34.2%) among Yemeni children aged ≤ 15 years in rural areas [3]. The current study is considered the first study to demonstrate the prevalence and risk factors of IDA among medical university students in Hodeida province, Yemen. Our findings showed the prevalence of IDA among medical university students (30.4%), of whom (54.00%) were female (*n* = 82) and (46.00%) were male (*n* = 70), which agrees with previous WHO's report that estimated the prevalence of anemia among females at reproductive age was more than 50% [15].

Possible causes of the high prevalence rate of IDA among females population may include inadequate intake of dietary iron, poor bioavailability, a concurrent inadequate intake of dietary micronutrients, lack of awareness of iron deficiency, and nutritional status. Initially females are more prone to be anemic than males particularly at reproductive age because of menstruation and due to socioeconomic customs; they get a diet of lower quality compared to males [16, 17]. Moreover, the higher prevalence of anemia among females in our population could be contributed to the traditional cultural practices in some families in which they tend to give more priority and more rights to the males than females especially in food sharing.

A previous study on Bengali University students revealed that 55.3% of students were IDA, of whom 63.3% were female and 36.7% were male, with high significant difference (*p* < 0.001) [6]. Another study reported in India showed that the prevalence of IDA among medical students was found to be 32.0%, of whom 44.0% were females and 20.0% males [18]. In addition, the prevalence of IDA among university students was found to be 23.9% in Saudi Arabia, 29.0% in United Arab of Emirates, and 3.8% of IDA in Iran [19–21], while the prevalence of IDA in this study was found to be 30.4%.

The high prevalence of IDA among adolescents may due to increase necessity of iron for rapid growth, menarche, and low intake of iron-rich food. Moreover, inappropriate dietary choices and frequently consumption of tea, coffee and cola with meals are associated risk factors for anemia [22].

The highest prevalence of IDA among our population could be linked to poverty which resulted in insufficient nutrition and inadequate health care as well as educated states [3]. Besides sex and age, this study investigated some possible risk factors significantly associated with IDA among the participants students as follows: the low-income families, no or infrequent intake of breakfast, red meat, fish, chicken, vegetables, and fruits, and some lifestyle habits (drinking tea, chewing khat, and smoking), and unawareness about anemia and its causes.

In our current study, regular breakfast intake revealed statistically significant (*p* < 0.001) difference among IDA (40.8%) than nonanemic students (76.7%). Similarly, students who consume irregular breakfast intake were demonstrated to have a higher IDA (59.2%) than nonanemic group (23.3%). Healthy breakfast food that contains heme and non-heme iron such as fat, meat, proteins, bread, fiber, grains, pulses, legumes, fruits, vegetables, minerals, and vitamins especially vitamin C is necessary to provide energy and enhanced iron absorption [23]. Earlier study on Bengali students revealed that, among anemic students, 41.0% regularly and 59.0% irregularly had breakfast intake compared to 68.7% regular and 31.3% irregular breakfast intake of nonanemic students [6]. Missing breakfast meals among university students might be due to low household income, waking up late, not being hungry in the morning, dislike of served food, or cutting calories to lose weight as in females.

The frequent (46.6%), infrequent (36.8%), or no (16.7%) consumption of vegetables and fruits; frequent (32.5%), infrequent (45.4%), or no (22.1%) consumption of red meat, fish, or chicken of nonanemic students compared to the frequent (18.4%), infrequent (50.7%), or no (30.9%) consumption of vegetables and fruits; frequent (18.4%), infrequent (36.2%), or no (45.4%) consumption of red meat, fish, or chicken of IDA students was statistically significant different (*p* < 0.001). Previous studies on Bengali students and Saudi women have demonstrated that low consumption of meat, vegetables, or fruits is associated with IDA [6, 24].

On the one hand, the current study revealed statistically significant difference (*p* < 0.001) between nonanemic and IDA students based on tea consumption, which agrees with previous studies reporting that the intake of tea was significantly higher among anemic subjects [25, 26], while disagreeing with another study where no association was found between the anemic and nonanemic subjects with respect to tea consumption [27]. On the other hand, the current study revealed that there were no statistically significant differences found with regard to coffee or cola consumption, while a previous study reported coffee consumption as a factor in iron deficiency anemia among pregnant women [28]. Moreover, another study reported that cola consumption significantly increased the risk of anemia, but no association was found with respect to coffee consumption [29].

The harmful effect of tea, coffee, and cocoa on anemia may be justified as they contain polyphenols (tannins) that inhibit absorption of iron from intestine [25]. Additionally, our students are not aware of foods and drinks that contain high amount of inhibitors which have an influence on iron absorption. These inhibitors are found in phytates (bread, wheat bran, breakfast cereals, oats, and rice); tannins or polyphenols (tea, coffee, cocoa, and certain vegetables); calcium (milk and cheese); and phosphate [30].

Several studies had been conducted about the association of chewing khat and higher prevalence of anemia, which could be explained by the loss of appetite [31]. Besides, khat contains a substantial amount of tannin, which reduces the bioavailability of non-heme iron from the diet that is mainly based on foods of plant sources in the population [32, 33].

Currently chewing khat has become an epidemic over East Africa especially in Ethiopia, Somalia, and South Arabia from the old to young, male and female, urban and rural settings as well as in our country. Furthermore, chewing khat has become a common practice among high school, college, and university students [34]. The association of IDA with chewing khat that causes the loss of appetite and reduces iron absorption is well documented [31]; the authors reported that the subject who chewed khat every day had a 29% higher risk of anemia than those who did so occasionally or never [31]. By contrast, findings of the present study showed that the prevalence of IDA (25.0%) was significantly lower among students who were chewing khat every day compared to nonanemic students (47.1%). Our study suggests that chewing khat every day leads to the insufficient intestinal absorption of bioavailable iron and then smoking could increase hemoglobin levels among nonanemic students who are chewing khat and smoking. In general, this study is in agreement with studies reporting that chewing khat may lead to loss of appetite which may cause a general malnutrition resulting in IDA. Also, the habit of chewing khat reinforces the appearance and the development of other habits like cigarette smoking and consuming plenty of liquids (cola, black tea, coffee, and cold water).

Smoking habit has been found to be associated with a higher prevalence of IDA in many studies [35]. Although cigarettes smoking causes increase of hemoglobin and hematocrit levels which could be related to the effect of exposure to carbon monoxide which reduces oxygen tension and causes hypoxia in the body [36]. Hypoxia consequently increases production of erythrocytes from blood forming organs and elevates levels of hemoglobin and hematocrit, while serum ferritin may be low [37]. However, the effect of smoking on other iron indices is not clear [36]. In this study, it was found that 21.8% of nonanemic students were cigarette smokers compared to 2.6% of IDA, with statistically significant association (*p* < 0.001). This could be explained by the chewing khat coexisting with smoking among our population.

Findings of the present study further showed that students of families with household monthly income < YER 50,000 (1 USD = 450 YER) had significant (*p* < 0.001) related factor which contributed to higher prevalence of IDA (34.2%) compared to their nonanemic couterparts (7.8%). Recently, Yemen is classified among the lower middle income countries with >50% of the population lives below the poverty line and had a very low purchasing power [10]. Low family's income and poverty had been documented in many studies as important factors provoking the prevalence of IDA and resulting in low overall food intakes and poor diets with low micronutrient content [38]. In Iran, 30–50% of women and children, especially those in low-income families, are suffering from iron deficiency [39–41].

## 5. Conclusion

IDA is highly prevalent and considered as serious health problems among university students, in Hodeidah province, Yemen. Our findings showed that more than half of the female students were found to be IDA than males. Most of cases IDA were occurring due to the lack of healthy iron-rich foods in daily food, drinking tea, irregular intake of breakfast, low household monthly income, and chewing khat, all of those were identified as the significant risk factors increasing the prevalence of IDA among university students. To prevent the prevalence of IDA among students, a proper health education to increase knowledge about anemia and its causative factors, benefits of taking iron-rich food, and avoiding unhealthy food and drink intake is needed.

## Figures and Tables

**Table 1:** Levels of Hb, MCV, SF, SI, and TIBC parameters and prevalence of iron deficiency anemia among Yemeni University students according to age and gender.

Parameters	Nonanemic students				IDA students				*p value*
	Male	%	Female	%	Male	%	Female	%	
Gender	256	78.5	92	54.0	70	21.5	82	46.0	<0.001

Age by years									
17–19	12	4.7	24	26.1	10	14.3	28	34.1	<0.001
20–22	189	73.8	54	58.7	45	64.3	45	54.9	
23–25	55	21.5	14	15.2	15	21.4	09	11.0	

Hb (g/dL) Mean ± SD	14.41 ± 0.84		13.10 ± 0.60		12.00 ± 0.80		10.82 ± 0.63		<0.001

MCV (fL) Mean ± SD	87.80 ± 6.20		88.44 ± 4.00		72.40 ± 4.45		72.30 ± 3.45		<0.001

SF (ng/ml) Median IQR	41.00 (33.63, 48.90)		34.50 (33.05, 37.30)		5.35 (3.65, 8.00)		5.8 (4.41, 7.93)		<0.001

SI (*µ*mol/L) Median IQR	31.95 (29.10, 33.03)		28.2 (25.03, 29.70)		11.20 (9.00, 12.00)		9.3 (8.10, 10.74)		<0.001

TIBC (*µ*mol/L) Median IQR	59.65 (47.65, 70.20)		74.6 (52.70, 76.30)		108.1 (96.75, 113.95)		111.0 (102.1, 115.84)		<0.001

IDA = iron deficiency anemia; Hb = hemoglobin; MCV = mean corpuscular volume; SI = serum iron; SF = serum ferritin; SD = standard deviation; IQR = interquartile rang.

**Table 2:** Distribution of anemic (IDA) and nonanemic students according to risk factors (social habits).

Variables	Answers	Nonanemic students (*n* = 348)		IDA students (*n* = 152)		Chi-square value	*p value*
		Total	%	Total	%		
breakfast intake	Regular	267	76.7	62	40.8	60.705	<0.001
	Irregular (nonregular)	81	23.3	90	59.2		

Vegetables and Fruits/week	No	58	16.7	47	30.9	37.235	<0.001
	Infrequently (<2 times/week)	128	36.8	77	50.7		
	Frequently (>3 times/week)	162	46.6	28	18.4		

Eating red meat, fish, chicken/week	No	77	22.1	69	45.4	29.131	<0.001
	Infrequently (<2 times/week)	158	45.4	55	36.2		
	Frequently (>2 times/week)	113	32.5	28	18.4		

Drinking cocoa after meal/week	Yes	81	23.3	32	21.1	0.299	0.585
	No	267	76.7	120	78.9		

Drinking coffee after meal/week	Yes	139	39.9	56	36.8	0.427	0.513
	No	209	60.1	96	63.2		

Drinking tea/day	No	102	29.3	08	5.30	73.993	<0.001
	within every meal	104	29.9	27	17.8		
	after every meal	102	29.3	60	39.5		
	Yes >4 caps	40	11.5	57	37.5		

Family income	Very good (>200,000 YER)	180	51.7	29	19.1	74.779	<0.001
	Good (50,000–200,000 YER)	141	40.5	71	46.7		
	Low (<50,000 YER)	27	7.8	52	34.2		

Diet (fitness)	Yes	29	8.3	12	7.9	0.027	0.869
	No	319	91.7	140	92.1		

Showing khat *(Catha edulis)*	Yes	164	47.1	38	25.0	21.510	<0.001
	No	184	52.9	114	75.0		

Smoking	Yes	76	21.8	4	2.6	29.040	<0.001
	No	272	78.2	148	97.4		

Being aware of anemia	Yes	138	39.7	39	25.7	9.064	0.003
	No	210	60.3	113	74.3		
